# Layer-by-Layer Assembly of Electroactive Dye/LDHs Nanoplatelet Matrix Film for Advanced Dual Electro-optical Sensing Applications

**DOI:** 10.1186/s11671-020-03442-6

**Published:** 2020-11-10

**Authors:** Sepehr Lajevardi Esfahani, Shohre Rouhani, Zahra Ranjbar

**Affiliations:** 1 Organic Colorants Department, Institute for Color Science and Technology (ICST), Tehran, Iran; 2 Surface Coatings and Novel Technologies, Institute for Color Science and Technology (ICST), Tehran, Iran; 3 Center of Excellence for Color Science and Technologies, Institute for Color Science and Technology (CECST), Tehran, Iran

**Keywords:** Solid-state sensor, Layer-by-layer (LbL), Electrochemical sensor, Optical sensor, LDHs, Alizarin reds

## Abstract

It proved that the most destructive effects of the toxic Al^3+^ ion on the human nervous system and disease that are involved with this system, such as Alzheimer's. The development of solid-state electrodes is still in its infancy during the sensor-based detection methods for Al^3+^. Hence, in this study, a novel flexible ITO/PET-based electrochemical solid-state sensor was designed and constructed. Modification of the surface of electrode bedding was done by layer-by-layer (LbL) assembly of Mg–Al LDH. nanoplatelets along with alizarin red S (ARS) in an interconnected matrix film. In the molecular design of sensing base of the electrode, the electroactive organic units (ARS molecules) present in the ITO/PET-layered (ARS/LDHs)_n_ matrix are involved in electrochemical reactions when exposed to the target molecule (Al^3+^ ion), so the electrochemical changes of the new formed Al-chelated system are detectable. This type of sensor is used for sensitive and selective detection of Al^3+^. The minimum sheet resistance, morphology and high electrocatalytic activity of the modified matrix film are obtained in the fifth cycle of LbL assembly technique. In this electrochemical sensor, both electrochemical and optical methods were detected with high sensitivity and selectivity of Al^3+^, so that in a cyclic voltammetry electrochemical method, the lower detection limit of 10.1 nM with a linear range of [0.2–120 μM] was obtained compared to the fluorescence-based optical method.

## Introduction

Layered double hydroxides (LDHs) are two-dimensional compounds of the general type of anion clay that are widely used in optical, biological and electronic applications [1–4]. The chemical structure of LDHs is introduced as [M^II^_1−*x*_M^III^_*x*_(OH)_2_]^−^(A^n−^)_*x*/n_. mH_2_O (M^II^ and M^III^ are divalent and trivalent metals, respectively, and A^n−^ is an inter-layered anion) [1]. Due to the two-dimensional structure, the flexibility and ionic exchange of LDHs, with the help of a new layer-by-layer (LbL) assembly method, combine this combination with a suitable organic molecule in a regular-layered arrangement with each other and form organic/inorganic compounds that have been extensively used in various fields of science [5–7]. One of these species as a guest molecule in this arrangement is the electroactive molecule, which results in accelerating the direct electron transfer process between the species involved in the electrochemical reaction and the initial electrode bedding [8, 9]. The use of LDHs nanoplatelets and structural reforms on their surface has found widespread use in the development of electronic and optoelectronic devices [10]. Some of the essential researches in this field are the following: Chen and colleagues [11], using a new and straightforward method, with single-stage Ni–Co LDHs hybrid film growth using ultrathin and porous nanosheets, could significantly increase the specific capacitance of the electrode based on this hybrid film, which compared to other similar electrode based on Ni-Co oxide/hydroxide was strongly enhanced. Li et al. [12] succeeded in intercalating of two kinds of anions with different molar ratios to the Mg–Al LDHs network and thereby obtain blue luminescent hybrid materials. Tian et al. [13] localized gold nanoclusters (Au NCs) inside the two-dimensional LDHs grid with layer-by-layer assembly to improve the fluorescence and efficient method to fabricate Au NCs-based films. Layered by layered assembly organization was considered a primary new method for fabricating fluorescence sensors with high fluorescence efficiency. Therefore, the precise molecular and structural design of the electrode base in an electrochemical device using advanced materials false detection and display of highly elegant electrochemical and optical changes within the designed network can significantly enhance the efficiency and sensitivity of the equipment.

Transparent electrodes have recently attracted more attention due to the critical components for optoelectronic devices. Recently, flexible and stretchable electronics and optoelectronics technology devices growing up fast, especially promoting the development of wearable electronics and highly portable features [14] Research on flexible electronics is rapidly expanding. It already exhibits applications in supercapacitors, implant sensors, flexible piezoelectric sensors, electronic paper, solar cells, touch panels, wireless wearable gadgets, flexible displays, bio-integrated therapeutic devices and epidermal electronics. Indium tin oxide (ITO) displays excellent optoelectronic features. It is at present the most widely used transparent electrode [15–17] Moreover, up to now ITO-coated polyethylene terephthalate (PET) substrates (ITO/PET) has been applied in numerous applications since they couple good flexibility and high electrical conductivity [18].

Recently, we reported an LDH base electrochemical sensor with dual amplification strategy for dopamine and Fe(III) detection. During the layer-by-layer (LbL) assembly, the synthesized electroactive naphthalimide dye/LDH nanoplatelets matrix (NALD-n) displayed in successive layers on the GO/ITO/PET surface electrode. This structure design resulted in a significant increase in the fluorescence emission of electroactive dye in the sensor's bed [19].

This field of molecular engineering in the structure of highly sensitive electronics, such as nanosensors, is the basis of the process designed in this research work.

Alizarin red S with the chemical name of 1,2-dihydroxy-9,10-anthraquinone has a flat heterocyclic ring structure, which has been widely used in biological sciences, photochemicals and environmental studies involved with toxic contaminants [20, 21]. ARS has been widely used as a sensor for the detection of metal ions and biomolecules because of high fluorescence intensity [22, 23].

Al^3+^ ion is a heavy and toxic metal ion that has numerous applications in various industries. Aluminium ion toxic and destructive effects on human health are investigated in several studies. The most destructive effects on the human nervous system, such as Alzheimer's reported [24]. Several precise laboratory methods are usually used to determine the amount of aluminium ion in biological and environmental samples. Some of these commonly used methods are including inductively coupled plasma atomic emission spectroscopy (ICP-AES) [25, 26], inductively coupled plasma atomic emission spectroscopy (ICP-MS) [27], graphite furnace atomic absorption spectroscopy (GF-AAS) [28], high-resolution continuum source flame atomic absorption spectrometer (HR-CS) [29], flame atomic absorption spectrometry (FAAS) [30]. These methods are standard and functional, but there are some serious disadvantages in serious matrix interferences, as well as the lack of accuracy. Different methods for aluminium ion determination are listed in Table 1. As can be seen, its detection limit is in the range of mg/l or µg/L. Some methods need a long time for sample preparation and analysis.

**Table 1:** Comparison of the analytical methods for the determination of aluminium ion

Method	Detection limit	Response time	Linear calibration graph	RSD (%)	Sample	References
ICP-AES	1.96 × 10^–3^ (mg/L)	–	–	3.7	Biological samples and	[25]
ICP-AES	6 × 10^–5^ (mg/L)	3 s	–	1.6	Human urine samples	[26]
ICP-MS	5 × 10^–5^ (mg/L)	–	–	11	Lake water	[27]
GF-AAS	9 × 10^–5^ (mg/L)	–	(1 × 10^–5^ to 250 × 10^–5^) × 10^–5^ (mg/L)	3.1–5.2	Biological and environmental	[28]
HR-CS	1.8 × 10^–4^ (mg/L)	–	(0.1 to 20.0) (mg/L)	2.4	Real water samples	[29]
FAAS	7.71 × 10^–3^ (mg/L)		(1 × 10^–3^ to 20 × 10^–3^) (mg/L)	5	Dam waters	[30]
Fluorescence	4.7 × 10^–7^(mol/L)	3 min	(6.19 × 10^–7^ to 6 × 10^−5)^ (mol/L)	< 5.0	Spiked lake and river water samples	[31]
Fluorescence	3.62 × 10^–6^ (mol/L)	40 s	(3.62 × 10^–6^ to 1 × 10^−4^) (mol/L)	2.82	Synthetic water	[32]
Colorimetric	1 × 10^–3^ (mg/L)	35 s	(0.1–1.0) × 10^–3^ (mg/L)	2.4–3.1	Synthetic water	[33]
Fluorescence	4.8 × 10^–12^ (M/L)	15 min	(1.0 × 10 ^−10^ to 1.0 × 10^–5^) (M/L)	< 5.0	Synthetic water	[34]
Reflectance	10^–5^ (mol/L)	5 min	(0.34 × 10^–3^ to 10.75 × 10^–3^) (mg/L)		–	[35]
Diffuse reflectance	6.7 × 10^–6^ (mg/ L)		(6.7 × 10^–6^ to 7.4 × 10^–6^) (mol/L)	8.8	Synthetic water	[36]
Fluorescence	1.1 × 10^–5^ (mg/L)	–	–	5	Dialysis solutions and water	[37]

In most cases, for trace analysis, preconcentration steps on a specific sorbent are needed. Some spectrofluorimetric methods show high sensitivity with low detection limit, but long response time [31–37]. Even though their detection systems were simple, but it shows weaknesses in sensitivity and selectivity. A combine optical and electrochemical methods sensor will offer better advantages over other methods. An optical–electrochemical sensor is compact for miniaturization, easily be incorporated into low-cost and easy-to-use with the excellent selectivity and sensitivity necessary for real-time environmental monitoring. Solid-state sensing platforms are essential in sensor fabrication. When a chemical reagent immobilizes into or onto a solid matrix, its stability improves in the immobilization media and so avoids the undesirable susceptibility to interference and fouling.

In the newer researches, the use of an electrochemical sensor is introduced for more precise detection of Al^3+^ ion [38]. A sensor that consists of a three-dimensional inorganic or two-dimensional organic/inorganic rigid network has a higher interfacial area for ion adsorption, and hence higher electrocatalysis reactivity in the sensor, which will result in higher sensitivity and selectivity of the electrochemical sensor [39]. Based on our knowledge, ITO/PET-layered (ARS/LDHs)_n_ matrix presented here is the first electro-optical platform for aluminium monitoring.

In this research work, a novel electrochemical solid-state sensor used to detect Al^3+^ ion accurately. The basis of this electrochemical sensor is the measurement of the signal by electrochemical and optical methods simultaneously. In this type of sensor, the electroactive organic units (ARS molecules) present in the indium tin oxide coated PET (ITO/PET) LbL assembled (ARS/LDHs)_n_ matrix are involved in electrochemical reactions when exposed to the target molecule (Al^3+^ ion), so the electrochemical changes of the new formed Al-chelated system are detectable. The electrochemical changes, the amount and quality of light released from the sensor are also assessed by contact with the target molecule.

## Materials and Methods

### Materials

ITO/PET sheet (60 Ω cm^−1^), alizarin red S (3,4-dihydroxy-9,10-dioxo-2-anthracensulphonic acid, sodium salt), aluminium(III) nitrate (Al(NO_3_)_3_), Cobalt(II) nitrate (Co(NO_3_)_2_), Ni(II) nitrate (Ni(NO_3_)_2_, Ca(II) nitrate (Ca(NO_3_)_2_), Sr(II) nitrate (Sr(NO_3_)_2_), Cu(II) nitrate (Cu(NO_3_)_2_), acetate/sodium carbonate buffers and potassium chloride (KCl) were obtained from Sigma Aldrich Co.

### Apparatus

The sheet resistance of modified electrodes was measured with Fluke Ohmmeter (1550B). Absorption measurements were taken with a single beam UV–Vis Spectrophotometer (CECIL CE9200) from 200 to 800 nm. Scanning electron microscopy (SEM) and atomic force microscopy (AFM) tests were done on prepared samples using (LEO 1455VP) and (Micro Photonics Inc/ Dual scope DS95-200E) instruments, respectively. A fluorescence spectrometer (PerkinElmer LS55) was used to measure the fluorescence intensity of tested samples. The cyclic voltammetry (CV) measurements were performed on electrodes using a (Zahner PP211 potentiostat) in the three-electrode electrochemical cell. In this test, the surface of prepared electrodes was used as a working electrode, and platinum was used as a counter electrode with Ag/AgCl as the reference one. A KCl solution (0.1molar in deionized water) was used as a standard electrolyte in all tests. In all of CV tests, the potential scan step was 0.005 V, and the potential scanning speed was 0.05 Vs^−1^. Each cyclic voltammogram presented in this research work is the most representative cycle obtained for each experiment.

### Fabrication of the A.R.S./LDHs Film Modified Electrode

**Fig. 1:**
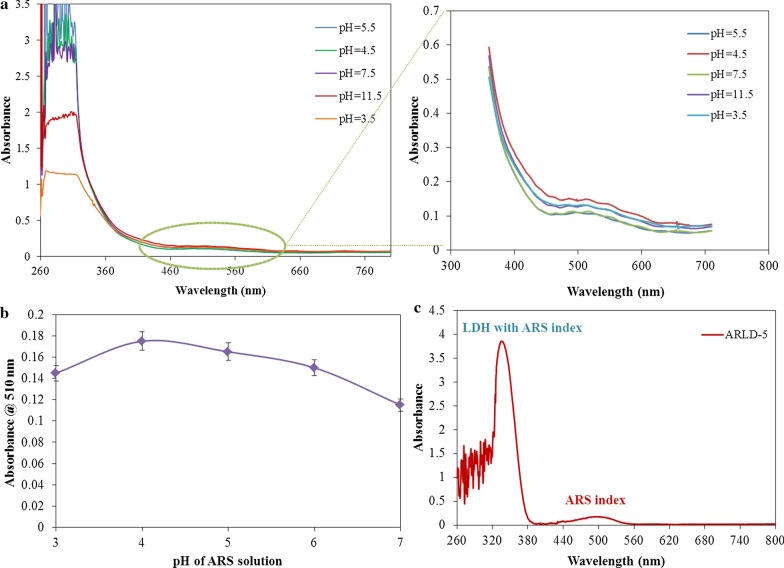
Absorbance changes of ARLD-n against pH **a** LDH colloidal suspension (1.0 g L^−1^) in deionized water in different adjusted pH (pH of ARS solution was fixed at 7 in LbL cycles), **b** ARS solution (1.0 g L^−1^) in deionized water in different adjusted pH (pH of LDH suspension was fixed at 5.5 in LbL cycles), **c** absorbance changes of ARLD-5 (pH of LDH suspension was fixed at 5.5 and pH of ARS solution was fixed at 4.0 through LbL cycles)

**Scheme 1:**
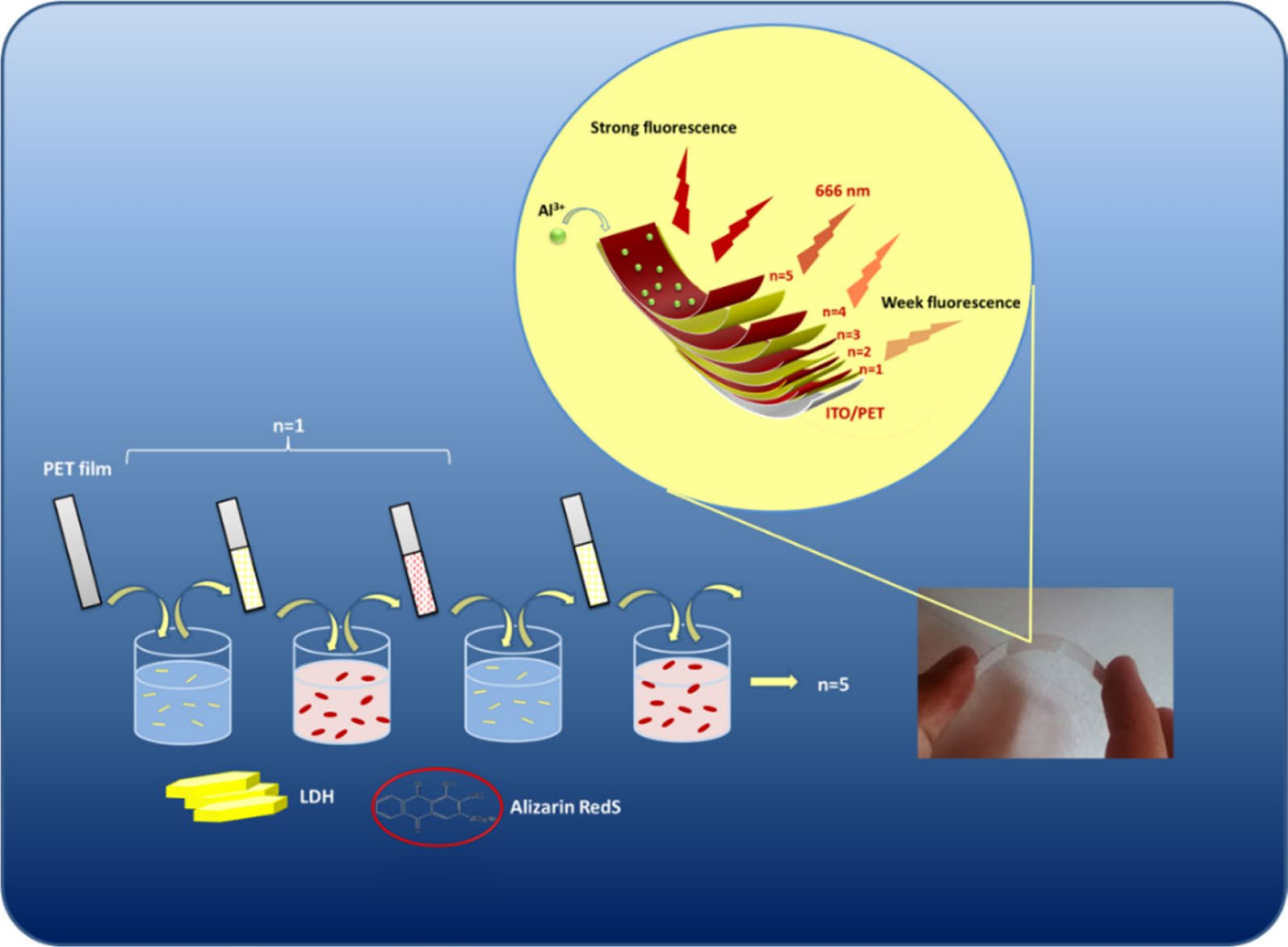
Schematic illustration for LbL assembly of LDH suspension (1.0 g L^−1^) and ARS solution (1.0 g L^−1^) to achieve modified electrode ARLD-5

Mg–Al LDHs (including nitrate anion) was synthesized by the method developed by the ICRC Research Group [40]. The colloidal suspension of Mg–Al L.D.H.s nanoplatelets in deionized water (1.0 g L^−1^) was prepared under ultrasonication in a sonicator bath (Bandelin electronic/510 h) at 40 °C for 4 h. In order to investigate the pH effect on the adsorption of LDH nanoplatelets and ARS dye molecules on the electrode surface in a range of pH values, which were adjusted by different (acetate/carbonate) buffers, the ITO/PET electrode was immersed in LDH suspension (in different adjusted pH varied from 3.5 to 11.5) for 10 min. The electrodes were then removed and rinsed with distilled water. Then they were immersed in ARS solution in distilled water (1.0 g L^−1^) with constant neutral pH (= 7.0). After immersion of electrodes in ARS solution, they were rinsed and dried with a nitrogen gas flow for 2 min. at 25 °C. This LbL process was repeated five times. UV–Vis spectrophotometry test was taken from the surface of the electrode after the 5th LbL cycle (Fig. 1a). Following the choice of optimal pH for LDH dispersion, the same tests were performed. This time, during one stage, the electrodes were immersed in LDH colloidal suspension (with optimum pH) for 10 min. After rinsing and drying, they were immersed in the ARS solution in different amounts of pH (other conditions were the same). The pH of the ARS solution was varied between pH 3.0 and pH 7.0. Then UV–Vis spectrophotometry test was taken from the surface of the electrode (Fig. 1b). Subsequently, a series of these operations for LDHs suspension (with optimum pH) and ARS solution (with optimum pH) were repeated n times to obtain multilayer films of (ARS/LDHs)_n_ (Fig. 1c). The procedure for LbL immersion tests is shown in Scheme 1. The characteristics of prepared electrodes with their codes are introduced in Table 2.

**Table 2:** The characteristics of prepared electrodes

Number of LbL cycles (*n*)	Sample code
*n* = 1	ARLD-1
*n* = 2	ARLD-2
*n* = 3	ARLD-3
*n* = 4	ARLD-4
*n* = 5	ARLD-5
*n* = 6	ARLD-6
*n* = 7	ARLD-7
*n* = 8	ARLD-8
*n* = 9	ARLD-9
*n* = 10	ARLD-10

## Results and Discussion

The effect of pH on the adsorption behaviour of LDH nanoplatelet was studied to obtain the optimum ARS adsorption on the solid-state electrode. UV–Vis spectrophotometry method was used for this. The LDH and ARS in the [260–360] and [400–600] regions show index absorption peaks, respectively [24]. The results of the pH optimization for LDH colloidal suspension and ARS solution are shown in Fig. 1.

As the results show, the minimum and maximum adsorption of LDH nanoplatelets with ARS molecules is observed in pH 3.5 and pH 5.5, respectively. Due to the isomeric displacement phenomenon in the LDH crystal network (replacing Mg^2+^ instead of Al^3+)^ and the charge level depends on the pH caused by the protonation/deprotonation on the LDH surface, metal ions of LDHs (in the basal surface of LDH) are dissolved in very acidic conditions (pH < 4) and the structure of the LDH is changed. The dissolution of LDH does not observe in other pH values [41–43]. The point of zero charges (PZC) is said to be the pH at which the opposite charges are zero under constant temperature, pressure and medium. Researches show that PZC has existed for LDHs [44]. The adsorption of proton charge on the LDH surface can alter the pH of the medium. However, charges on the surface of the LDHs can change with changing the pH of the system. Also, the arrangement of LDH nanoplatelets (basal and prismatic surfaces) due to the effect of electrostatic bonding in different pH conditions has different tendencies for adsorption of dye molecules. According to the results, in severe acidic conditions (pH < 4), due to the dissolution of metal ions on the basal surface of the LDHs, the amount of stable and unsolvable LDH nanoparticles that adsorb on the solid electrode surface will be minimal.

**Fig. 2:**
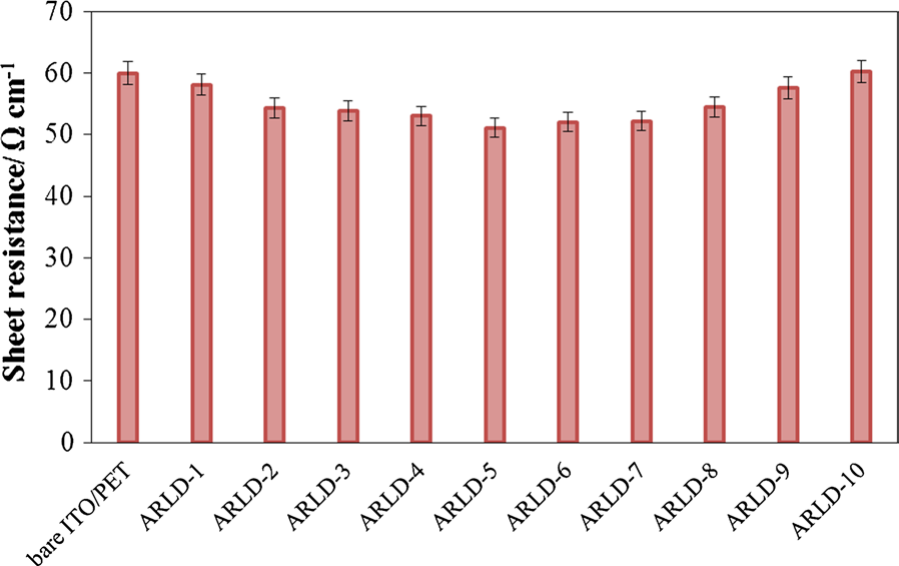
The sheet resistance changes of ITO/PET electrode samples layered by (ARS/LDHs)_n_

**Scheme 2:**
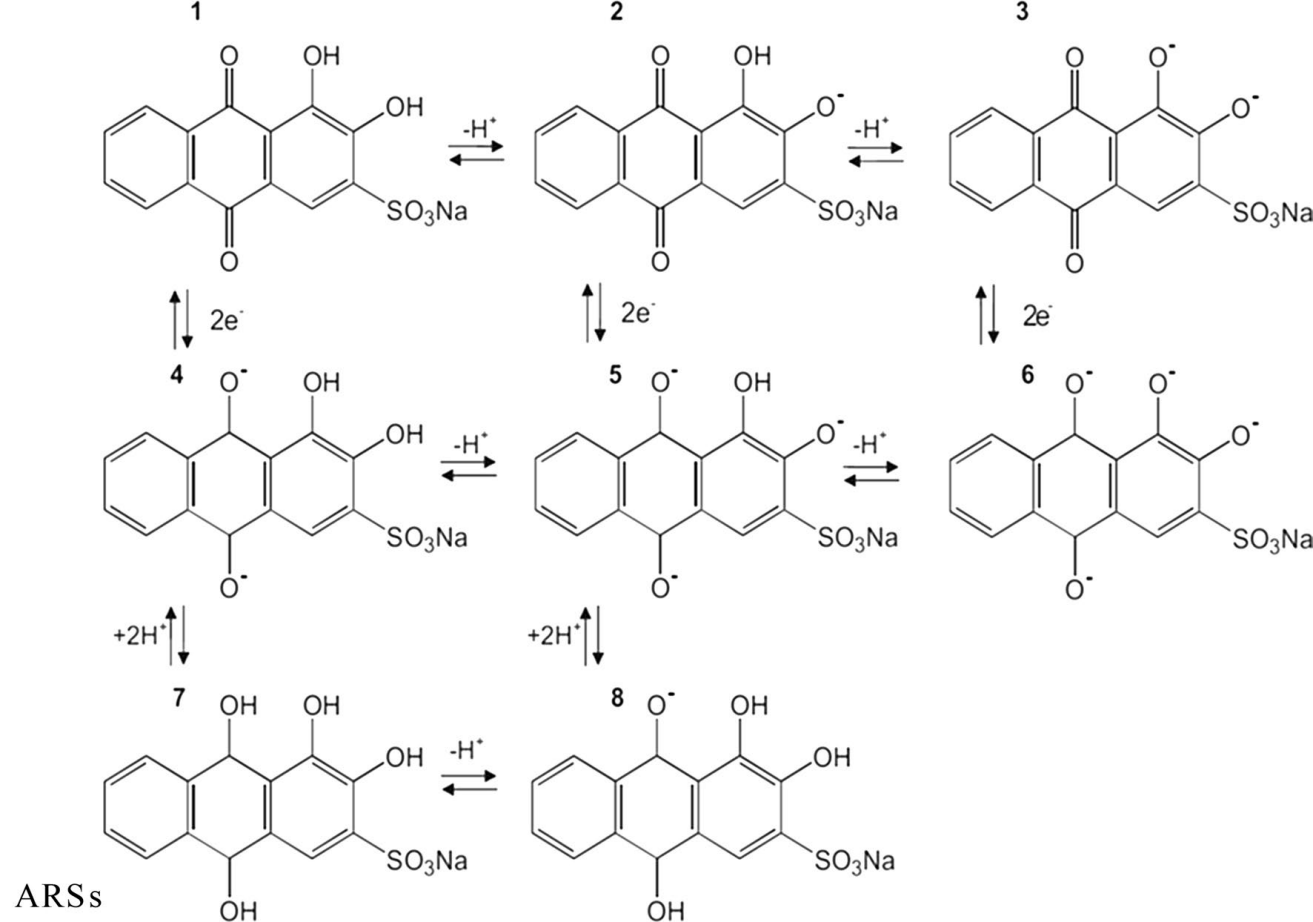
Equilibrium structures of ARS depending on the pH and tautomeric structures

On the other hand, by moving to entirely alkaline conditions, due to the deprotonation of the LDH, the lower amount of ARS molecules are adsorbed on the surface of LDHs. Therefore, the optimum pH of LDH colloidal suspension for LbL immersion test is considered to be 5.5. According to Fig. 2b, the highest adsorption of ARS on the electrode surface containing LDH nanoplatelets indicated in pH 4.0. It seems that an inappropriate mediator formed for formation of a complex with Al^3+^ ions on the surface of LDHs. Due to the significant acidic conditions (pH 4.0), the structural form of the intermediate compound (compound II in Scheme 2) is more stable, which is more stable conditions, can form a chelate with Al^3+^ ions in the LDHs network. As far as neutral pH is concerned, the relative adsorption of ARS on the LDH surface decreases. Sathish [45] and Supian et al. [24] also reported that ARS reagent formed a complex with an Al^3+^ ion in the acetate buffer at pH 4.0. Therefore, the optimum pH of ARS solution for LbL immersion test considered to be 4.0. The absorbance behaviour of ARLD-5 modified electrode can be seen in Fig. 1c, while the optimum pH adjustment was made for LDH suspension (pH 5.5) and ARS solution (pH 4.5) during all of five LbL cycles.

Regarding the effect of Mg–Al LDHs nanoplatelets on the electrical conductivity of the electrode surface, the number of immersion cycle (*n*) should be determined. For this purpose, changes in electrode sheet resistance were investigated in different immersion cycles. Figure 2 shows the results of the sheet resistance of ITO/PET electrode samples layered by (ARS/LDHs)_n_.

As the result of Fig. 2 is clear, the electrode's sheet resistance to the fifth cycle decreases slightly, and after the fifth cycle, the increasing sheet resistance is due to the excessive increase in LDHs nanoplatelets densities and the increase in the length of the electron transfer pathway. It is important to note that the LbL assembly of LDHs nanoplatelets on the electrode surface can result in the ionic exchange function and the robust electron transfer. Therefore, the fifth cycle was selected as the optimal cycle, so that the modified electrode in these conditions has the least resistance and the highest electrical conductivity. In general, the results of this test show that the LbL assembly method of LDHs nanoplatelets with the ARS dye molecules does not have a significant effect on the resistance and electrical conductivity of the electrode until the tenth cycle.

**Fig. 3:**
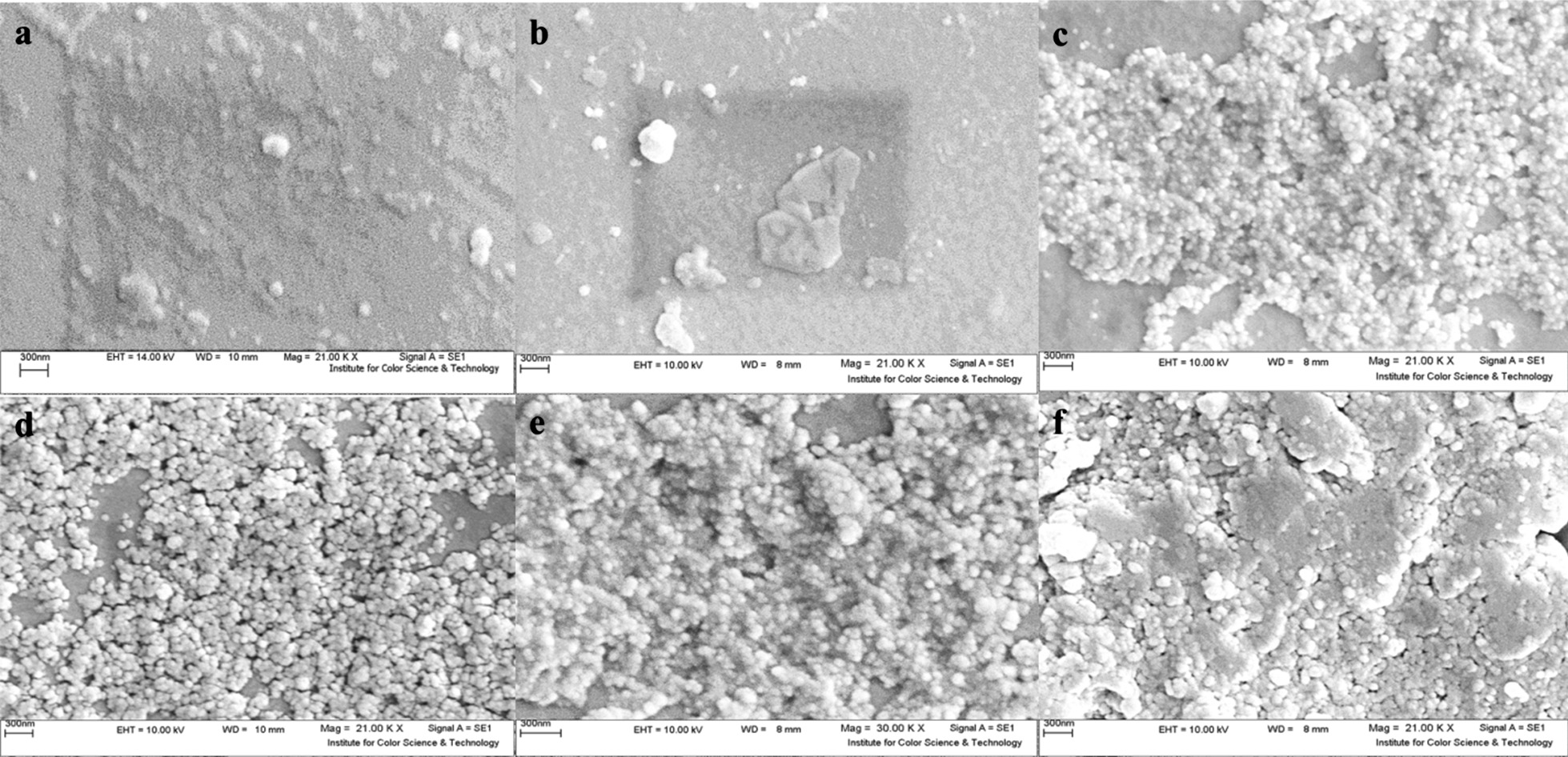
SEM images (top-view) obtained from the prepared solid-state electrodes (**a**: ITO/PET, **b**: ARLD-1, **c**: ARLD-2, **d**: ARLD-3, **e**: ARLD-4, **f**: ARLD-5)

**Fig. 4:**
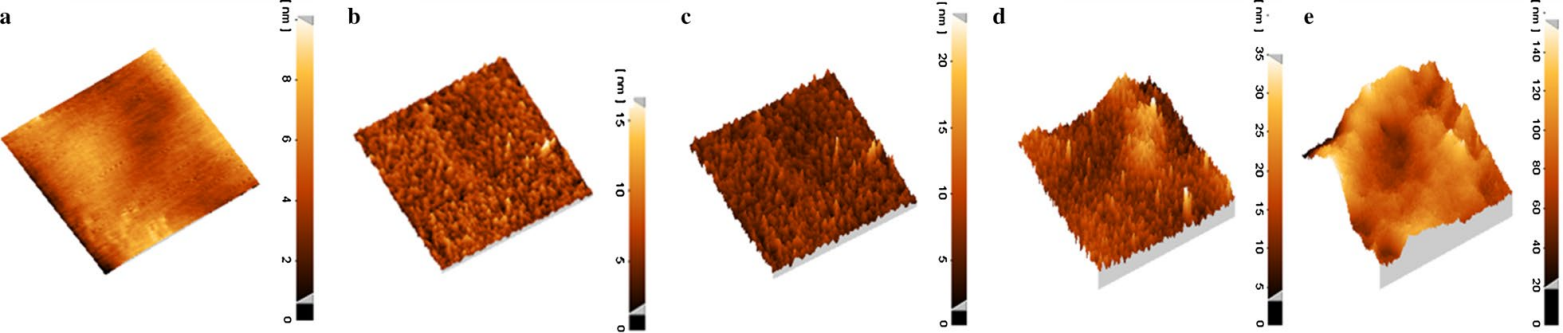
3D-dimensional topographic AFM images obtained from the prepared solid-state electrodes (**a**: ARLD-1, **b**: ARLD-2, **c**: ARLD-3, **d**: ARLD-4, **e**: ARLD-5)

In Figs. 3 and 4, SEM images and topographic AFM results obtained from the samples are presented, respectively.

As an illustration in the results of morphology and topography scanning of the prepared electrodes, with increasing the number of LbL cycles (*n*), continuous increase in the density of LDH layers along the ARS is observed on the surface of the electrode. As from the first cycle to the fifth cycle, surface roughness was increased (AFM images) and the thickness of the LDH nanoplatelets along with the adsorbed ARS continually was increased sharply.

**Fig. 5:**
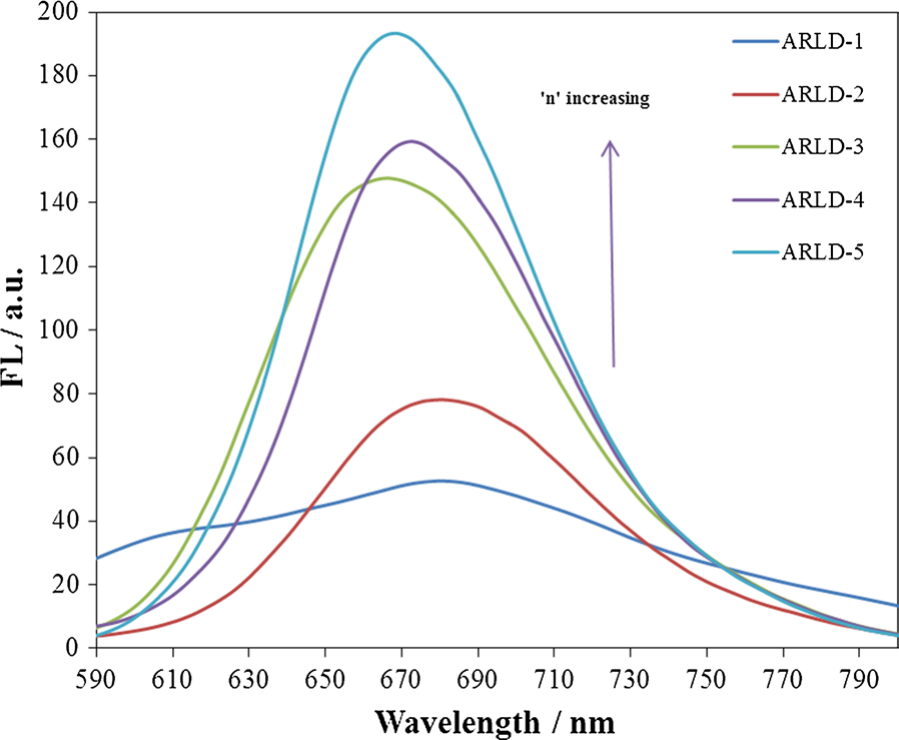
The changes in the fluorescence intensity of different prepared electrodes (*λ*_exc._ = 388 nm)

In order to investigate the adsorption of ARS molecules on the LDHs nanoplatelets, the optical behaviour of the adsorbed layers was studied using the spectrofluorimetry test. After each LbL immersion cycle, the spectrofluorimetry test was performed from the solid electrode surface. The results of the spectrofluorimetry test of the prepared electrodes are given in Fig. 5.

As the results show, after the first LbL immersion cycle, due to the lower amount of ARS molecules along with LDHs nanoplatelets adsorbed on the electrode surface, the amount of fluorescence emission is meagre and the peak of the weak fluorescence emission is about 666 nm. By increasing the number of LbL immersion cycles (*n*), the fluorescence emission rate increases continually by increasing density of ARS layers along with LDHs nanoplatelets on the solid electrode surface. As in the fifth cycle, the highest fluorescence emission peak is observed at 668 nm. Due to the weak emission spectrum of ARS solution in deionized water in low concentration (10^–4^ M), the fluorescence emission intensity of this compound when enclosed between Mg–Al LDHs on solid electrode substrate has improved due to less molecular rotation of dye molecules in solid-phase than dye molecules in solvent phase and reduced solvent effect in solid phase [46]. As the number of layers increases, the shape of the couriers will be sharpened, and the peak width will be lower, which confirms this. Fluorescence emission of molecules in aggregates forms or assembled into crystalline lattices undergoes enhancement, the effects of non-radiative energy transfer and reduction of molecular rotations are considerably reduced or eliminated. However, high aggregation on the solid surface resulted in a remarkable self-quenching. To reduce the intermolecular interaction between the chromophores, an increasing separation between stacked structures to reduce π-overlapping between aromatic cores, led to a reduction of fluorescence quenching and strong emission resulted [47].

It found that ARS has three equilibrium structures depending on the pH of the solution, as shown in Scheme 2 [48, 49]. The second deprotonation leads to a bluish violet dianion (III) at pH > 12.1. The species of oxidized ARS 1 and reduced ARS 7 present as a function of experimental conditions. As can be seen from below pH 4 ARS 1 represents the main species, ARS 2 prevails in the pH region of 5.5–10.5 and ARS 3 will be the relevant species above pH 12. Ionic forms of ARS have tautomeric structures. Scheme 2 shows the reduction forms and tautomerism of each species.

**Scheme 3:**
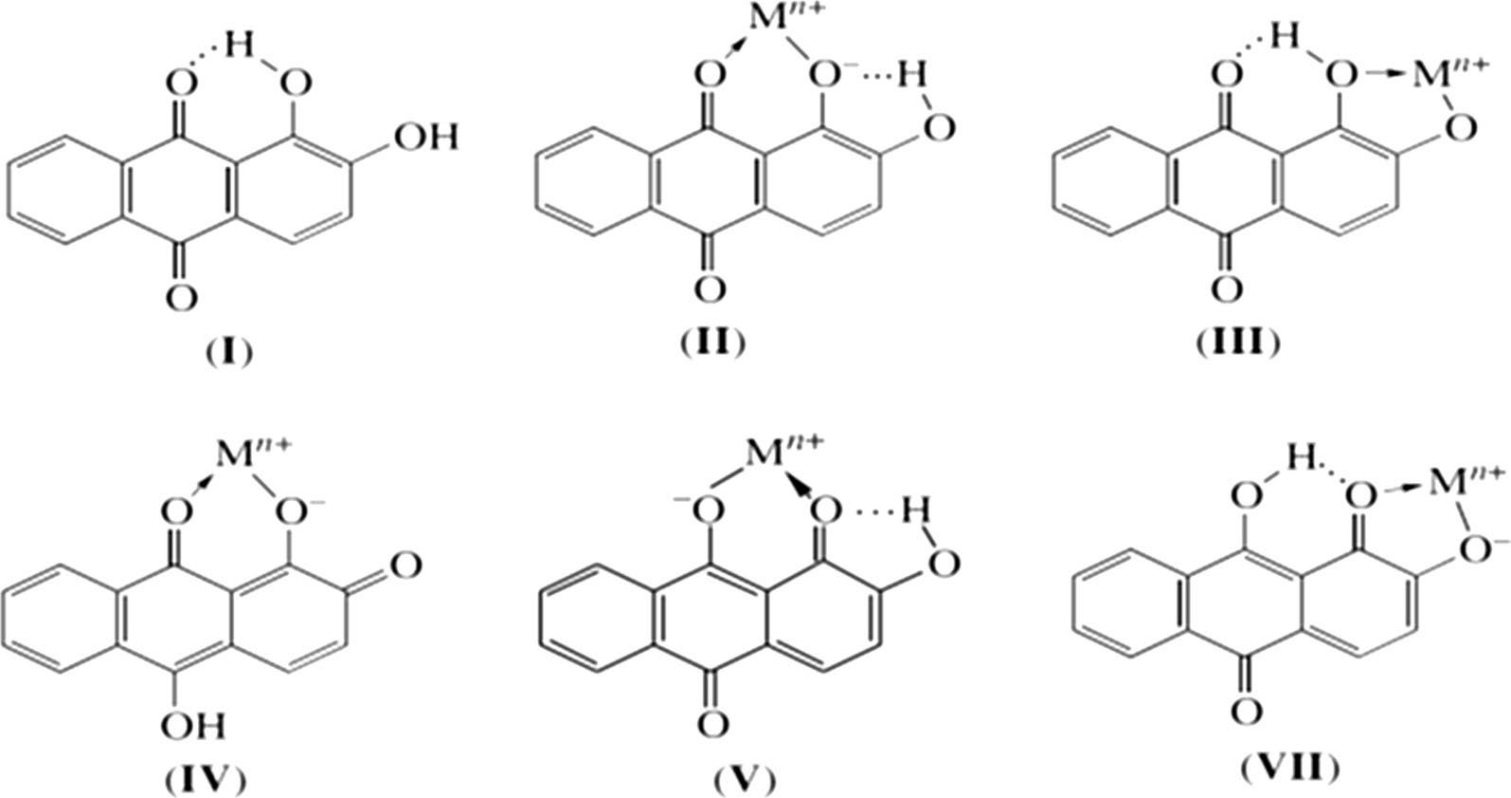
Tautomeric forms of ARS-Al complex

Alizarin (I) and ARS is known to form stable complexes with Al^3+^, known as “lake pigments”. V. Ya. Fain and coworkers deeply studied on metal complexes of alizarin and alizarin reds based on their electronic absorption spectra [50]. They concluded the complexation of dihydroxyanthraquinones always proceeds at the peri- or ortho-hydroxycarbonyl group and involves tautomeric anthraquinoid forms. Alizarin complexation occurs at the peri-hydroxycarbonyl group via the formation of the C=O M–O coordination bond and six-membered chelate ring in monoanionic form of ARS, compound II (Scheme 3). The authors suggested that in alkali solutions, alizarin reacted with metal ions as the ortho-diphenol compound to give structure III. Complexation in neutral media can be explained by the replacement of the hydrogen atom in the chelate cycle of complex I by the metal ion followed by the ionization of the M–O bond. Therefore, the ionized hydroxy group of alizarin monoanion in position 1 is bonded through the intramolecular hydrogen bond. Also, to form II, this complex can have three more tautomeric forms (IV–VI).

In acidic media, the O–M bond remains covalent, and the electronic absorption spectra of monometallic alizarinates are identical to the spectrum of nonionized alizarin. In weakly alkali media, alizarin enters complexation reaction with metal ions as monoanion. Of all monoanionic structures, only 9-hydroxy-2-oxo-1,10- anthraquinone contains the adjacent carbonyl and oxo groups. Therefore, monometallic alizarinates formed in an alkali medium should have the 1,10-anthraquinoid structure VII with the five-membered chelate cycle.

As can be seen in Scheme 2, ARS has a reversible redox behaviour. Its metal complexes are also electroactive with different redox characteristics, make it a right candidate for application in the electrochemical-based analysis. Its complexation rate is fast respect to some common metal ion complexing reagents. ARS and ARS-Al show different redox peak potentials. Therefore during the addition of aluminium ion to free ARS a new peak (ARS-Al) in different potentials related to ARS-Al redox is growing up proportional to aluminium ion concentration. Different stable platforms introduced in order improved the sensitivity and applicability of ARS-based sensors. Both ARS and its complex are electroactive in the solid state.

**Fig. 6:**
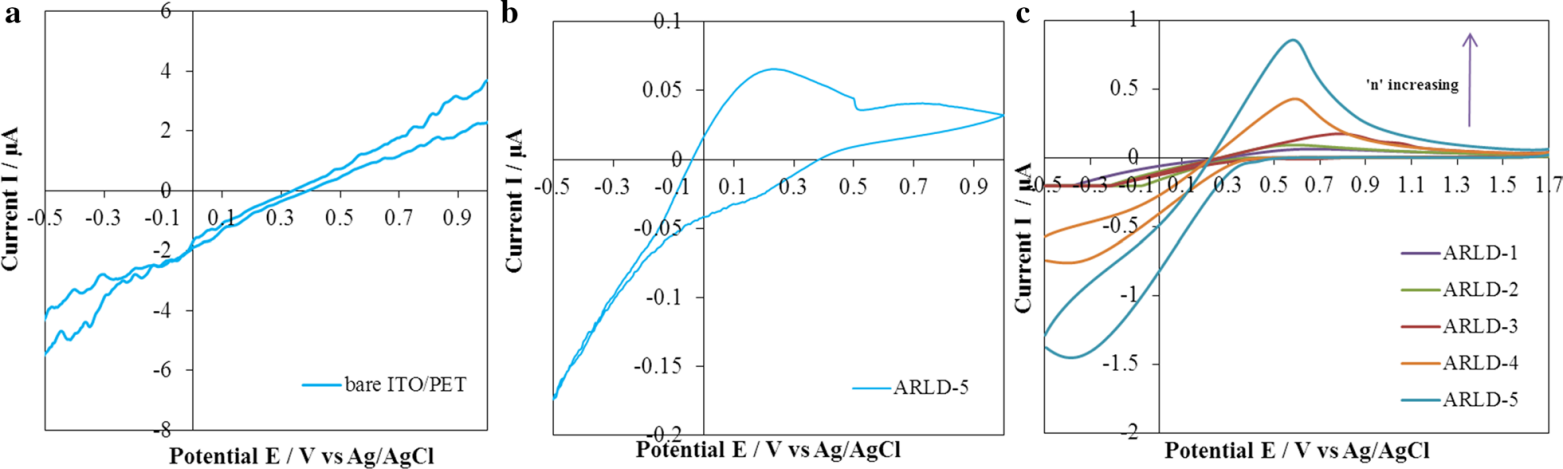
The cyclic voltammograms of **a** bare ITO/PET in 0.1 M KCl in deionized water, **b** ARLD-5 electrode in 0.1 M KCl in deionized water, **c** prepared electrodes in (0.1 M KCl in deionized water) including 15 μM Al^3+^

Electrochemical behaviours of prepared electrodes were studied by cyclic voltammetry test. The results of cyclic voltammograms of prepared electrodes are shown in Fig. 6.

According to the results of Fig. 6a, the shape of the bare ITO/PET electrode is a noisy graph with low current flow and a shallow width, indicating weak electrocatalytic activity of the electrode. With the modification of the electrode surface by the (ARS/LDHs)_n_ LbL assembly, the shape of the CV graph tends to wider and symmetrical cycles, so as the number of immersion cycles increases, the flow interval increases and the courier surface becomes more expansive, with the highest peak flow rate and the broadest graph in the fifth cycle (*n* = 5). As shown in the results of Fig. 6b, for the ARLD-5 electrode in the 0.1 M KCl, when no aluminium ion is present, a somewhat “reversible” peak is observed by the result of the CV test. In this peak, the anode and cathode currents in about 0.25 V (vs Ag/AgCl) occurred due to oxidation/reduction of free ARS on the modified electrode surface. In the presence of aluminium ions (Fig. 6c), a pseudo-reversible peak burst is detectable at about 0.58 V (vs Ag/AgCl), which is related to oxidation peak for the alizarin–aluminium complex on the modified electrode surface, so that in ARLD-5, oxidation peak can be detected sharply and distinctly. The results of the voltammogram show that, with an increasing number of LbL immersion layers, the electrocatalytic activity of the modified electrode has improved concerning the electron transfer to the external surface of the electrode. Also, the following reactions for the reduction of the (ARS-Al) complex (Scheme 3) on the electrode surface have improved, and the best electrocatalytic activity is evident in ARLD-5 electrode [24].

Regarding the importance of the pH effect on the structural model of dye and formation of an (ARS-Al) complex, all CV experiments were carried out in the neutral pH (= 7.0) (0.1 M KCl in deionized water), that all electrochemical and optical changes in the electrode surface can be controlled and detected only by changing the concentration of Al^3+^ ion.

### Electrochemical Detection of Al^3+^

**Fig. 7:**
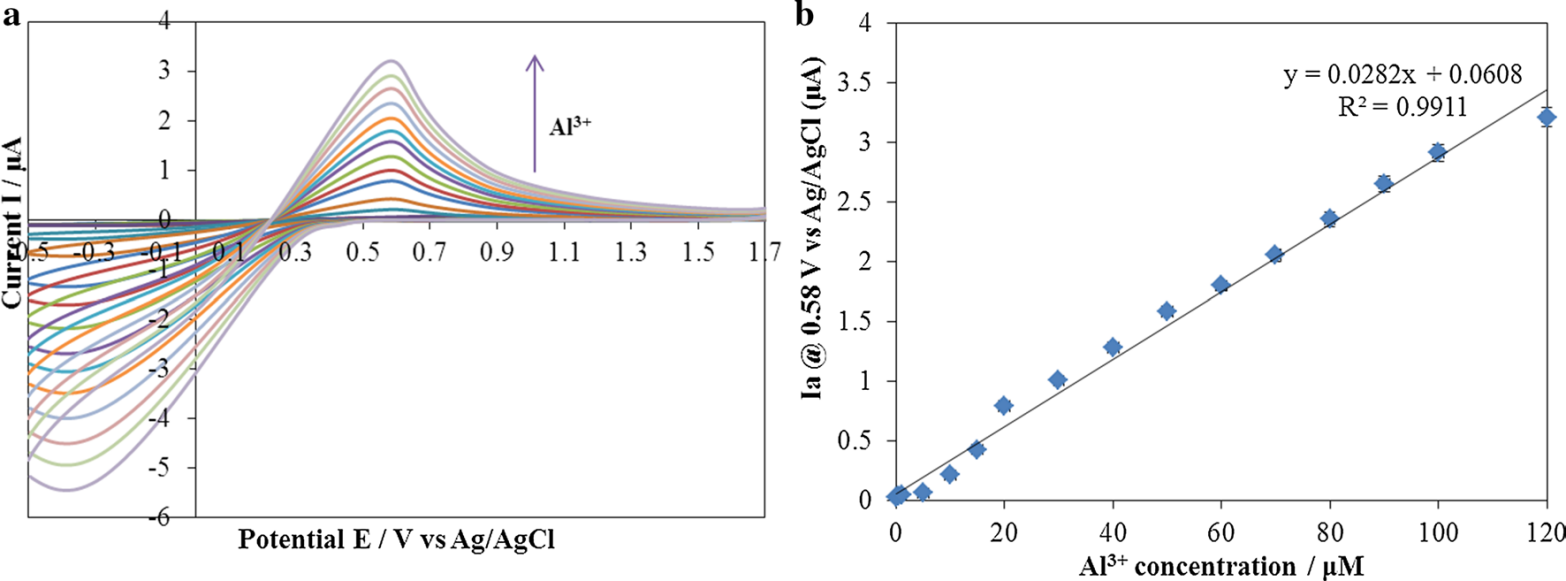
**a** The cyclic voltammogram of ARLD-5 electrode after adding different concentrations of Al^3+^ [Al^3+^ concentration range 0.2–120 μM], **b** the peak current changes of ARLD-5 electrode with different concentrations of Al^3+^

The cyclic voltammogram changes after adding different concentrations of Al^3+^ are shown in Fig. 7. All results are obtained after 10 s from the addition of aluminium ion to the electrolyte.

According to the results of Fig. 7, by increasing the concentration of Al^3+^ ion, the intensity of the oxidation peak current gradually increases at a peak potential (*E*_pa_) of 0.58 V (vs Ag/AgCl) with a controlled and continues process. It indicates the formation of ARS-Al^3+^ complex on the surface of the ARLD-5 electrode with the oxidation potential of about 0.58 V (vs Ag/AgCl). Due to the high strength of formation of the complex between A.R.S. and Al^3+^, the oxidation peak of the complex formation is pseudo-reversible [51]. The results obtained in this study are close to the results of other studies as the linear sweep scan shows the large peak at 0.27 V corresponds to oxidation of free alizarin while oxidation of Al complexed alizarin gives the peak at 0.58 V in different scan rates [52]. Figure 7b shows the electrocatalytic behaviour of the ARLD-5 electrode for the detection of Al^3+^ ion. The anodic peak current changes enhance linearly along with the increase in Al^3+^ concentration. The linear response ranges in [0.2–120 μM] with a regression equation of *i*_pa_ (μA) = 0.0282C (10^−6^ M) + 0.0608, *r*^2^ = 0.991, and a detection limit of 10.1 nM for Al^3+^ was obtained.

**Fig. 8:**
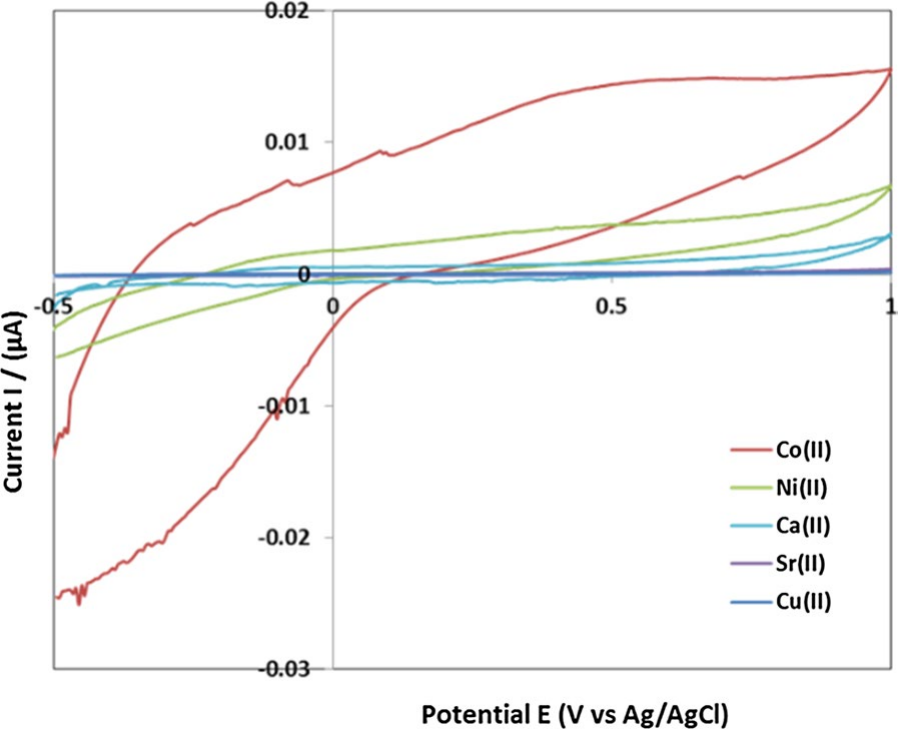
The cyclic voltammograms of ARLD-5 electrode after adding interfering ions

Furthermore, Co^2+^, Ni^2+^, Ca^2+^, Sr^2+^ and Cu^2+^ as interfering ions were investigated under the same conditions (ARLD-5 as working electrode, concentrations of 120 μM of interfering ions in 0.1 M KCl solution for testing CV). The cyclic voltammograms of ARLD-5 electrode after adding interfering ions are shown in Fig. 8.

**Fig. 9:**
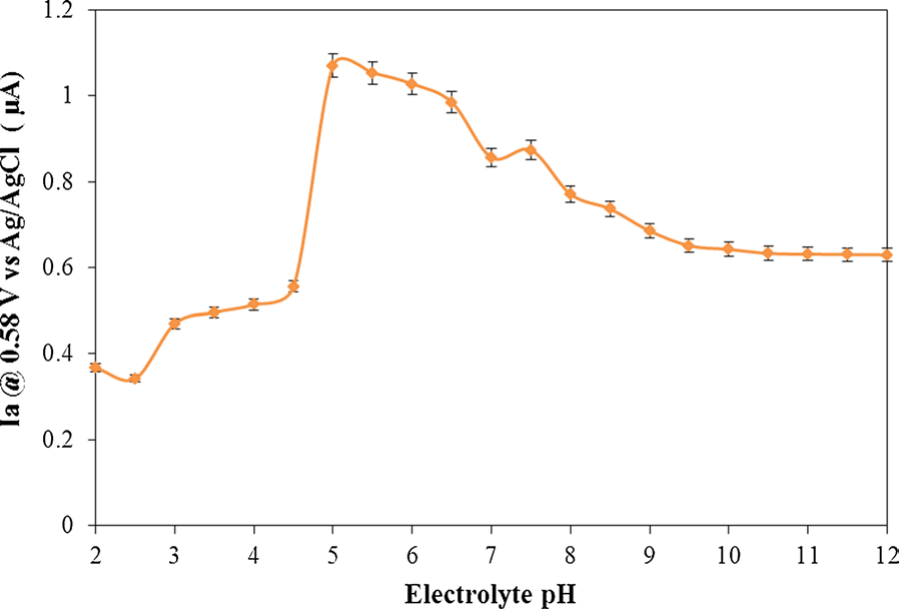
Anodic current changes at 0.58 V versus Ag/AgCl of ARLD-5 electrode to pH of 0.1 M KCl in deionized water (including 15 μM Al^3+^ ion)

Figure 8 demonstrates the good selectivity to Al^3+^ for the modified electrode. pH was changed by acetate/sodium carbonate buffers, 0.1 M KCl (including 15 μM Al^3+^ ion) from 2.0 to 12.0. The results of the cyclic voltammograms are shown in Fig. 9.

**Scheme 4:**
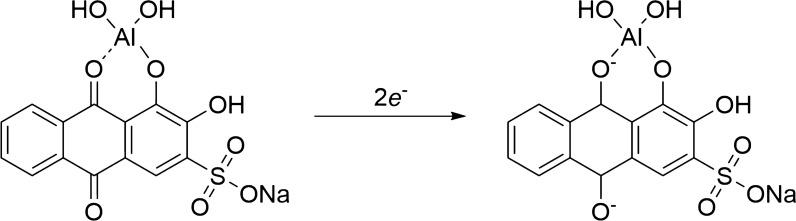
Mechanism of ARS complex reduction on the electrode surface

As shown in Fig. 9, in the pH range 5.0–7.5, there is little difference in the shape of the graph and the oxidation peak current. However, in more acidic conditions (pH range 5–6.5), due to the presence of higher hydrogen ion in the electrolyte, the probability of the formation of the compound II (in Scheme 4) on the surface of the solid electrode increases to form the chelate with the aluminium ion, and therefore, the oxidation peak current of the ARS-Al complex increases [38]. The application of this sensor is not recommended for pH less than 5.0 (severe acidic conditions) and more than 8.0 (severe alkaline conditions) due to the structural changes in adsorbed LDHs nanoplatelets on the electrode surface.

### Optical Detection of Al^3+^

Alizarin red S (ARS) has been used as a chromogenic agent for developing spectrophotometric methods. Anthraquinone-metal complexes show strong fluorescence. The fluorescence of metal–ARS complexes is affected by cation's field energy [53]. Therefore, the maximum excitation fluorescence wavelength pair of the Al-ARS complex is significantly different from other metal ions, which are interferents in spectrophotometric methods for Al determination. The principle of spectrofluorimetric determination of aluminium is based on its intensity enhancement which growing up during addition of aluminium ion to free ARS. Herein ARS was adsorbed on a solid platform of LDH, making it more rigid and improved its intensity resulted in more sensitivity.

**Fig. 10:**
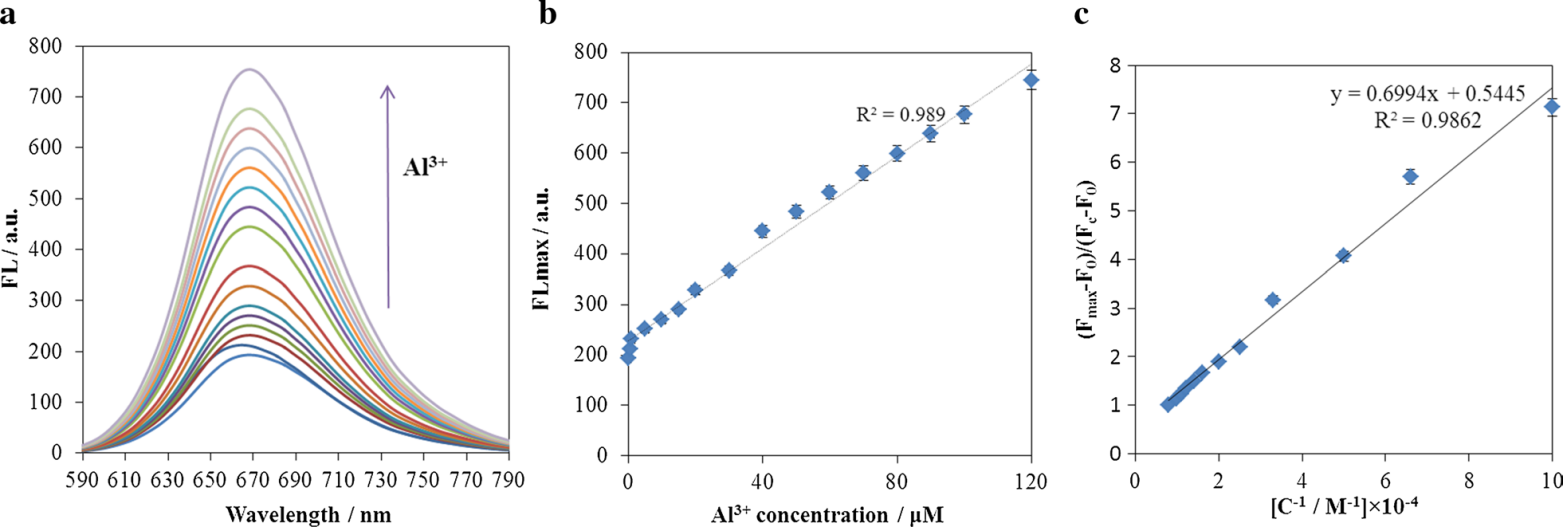
**a**, **b** The changes of fluorescence intensity (*λ*_exc._ = 388 nm) of ARLD-5 electrode after adding different concentrations of Al^3+^ [Al^3+^ concentration range 0.2–120 μM], **c** the fluorescence intensity changes in terms of Al^3+^ concentration inverse

Prepared electrodes were considered as the working electrode in the three-electrode electrochemical cell. The electrolyte used was a KCl solution (0.1 M) in distilled water. Different concentrations of aluminium ion were added to the electrolyte. After each CV test and completion of this test, the spectrofluorimetry test was regularly performed on the electrode surface. The results of spectrofluorimetry test are shown in Fig. 10.

The modified ITO/PET electrode with (ARS/LDHs)_5_ matrix shows an intense fluorescence emission spectrum in the presence of Al^3+^ ion. Figure 10 shows the gradual fluorescence increase in ARLD-5 after the addition of Al^3+^ (concentration range from 0.2 to 120 μM) and indicates the formation of a static adduct between A.R.S. and Al^3+^. It was found that the ARLD-5 fluorescence intensity increased within 10 s after addition of Al^3+^. So, the fluorescence increasing of ARLD-5 was relatively independent of the incubation time. From the results above, the ARLD-5 has a sensitivity and high-efficiency fluorescence emission to Al^3+^ ion.

To calculate the detection limit and calibration curve in an optical sensor, first, we obtain the ratio of the fluorescence intensity differences, draw then the graph of this ratio in terms of the target concentration inverse. According to Eq. (1) [54], the slope of the fitted line was used to determine the detection limit. The obtained results are shown in Fig. 10c.

1 $$(F_{\max } - F_{0} )/(F_{{\text{c}}} - F_{0} ) = 1 + ({\text{KC}})^{ - 1}.$$

As the results of Fig. 10b shows, the fluorescence intensity changes enhance along with the increase in Al^3+^ concentration with the linear response ranges of [0.2–120 μM]. Plotting fluorescence intensity versus inverse of the concentration, the regression equation of *Y* = 0.6994*X* + 0.5445, *r*^2^ = 0.986, and a detection limit of 23 nM for Al^3+^ was obtained.

**Fig. 11:**
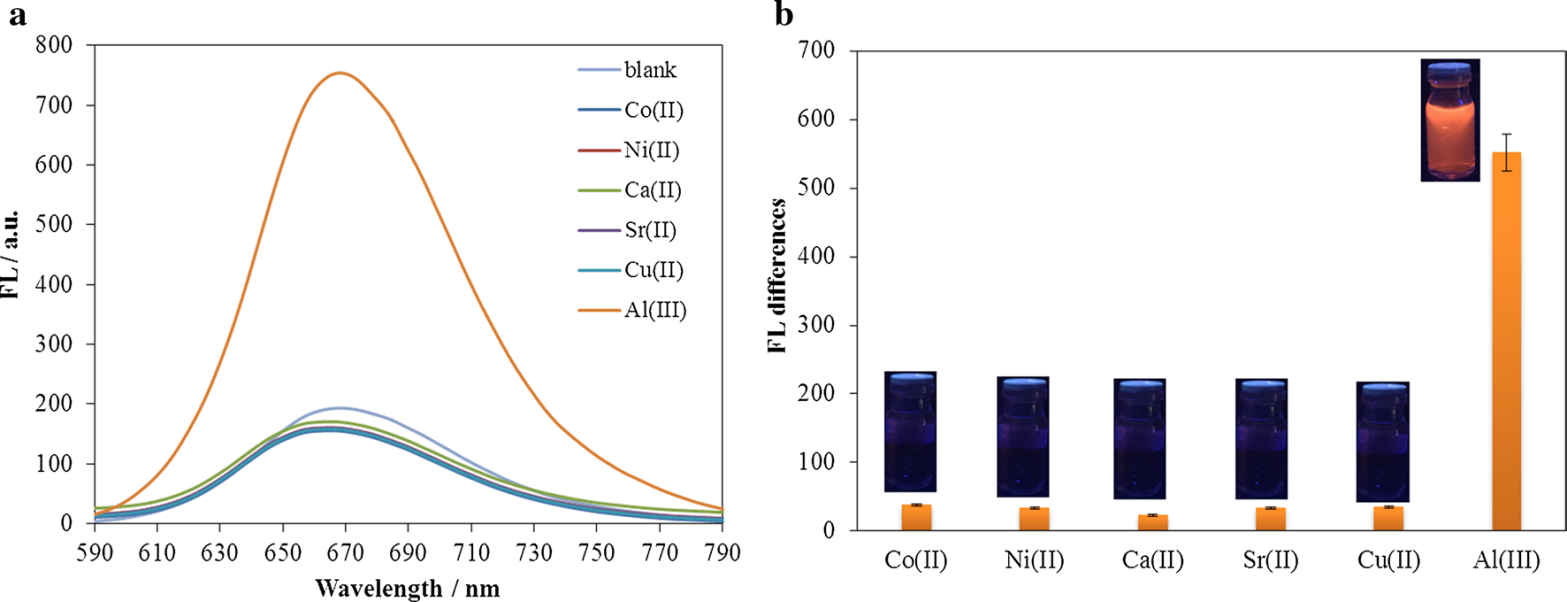
**a** The fluorescence intensity (*λ*_exc._ = 388 nm) changes of ARLD-5 electrode after adding 120 μM of Al^3+^ and interfering ions, **b** the fluorescence intensity differences for Al^3+^ detection compared to interfering ions

In order to determine the selectivity of the sensor to the Al^3+^, a concentration of 120 μM interfering ions (Co^2+^, Ni^2+^, Ca^2+^, Sr^2+^, Cu^2+^) were prepared. The spectrofluorimetry test was carried out from the electrode surface after adding the interfering ions to the electrolyte. As shown in the results of Fig. 11, it is clear that the addition of interfering ions did not show a significant difference in the fluorescence emission of the electrode surface, and the most significant difference was observed in the emission of fluorescence for Al^3+^, indicates the high selectivity of this type of sensor for Al^3+^. In order to better determine the selectivity of the modified electrode to Al^3+^, six solutions of 10^–4^ M concentrations of ARS in distilled water were prepared in completely same conditions. Therefore, 120 μM of Al^3+^ ion and interfering ions were assigned to each of the solutions and mixed well. Figure 11b shows the images taken under UV cabin with an excitation wavelength of 366 nm of each of the solutions. As the spectrofluorimetry test results showed, adding Al^3+^ ion to ARS solution showed significant fluorescence, while the initial solution of ARS and other ARS solutions contained interfering ions under the UV cabin have no fluorescence emission.

**Fig. 12:**
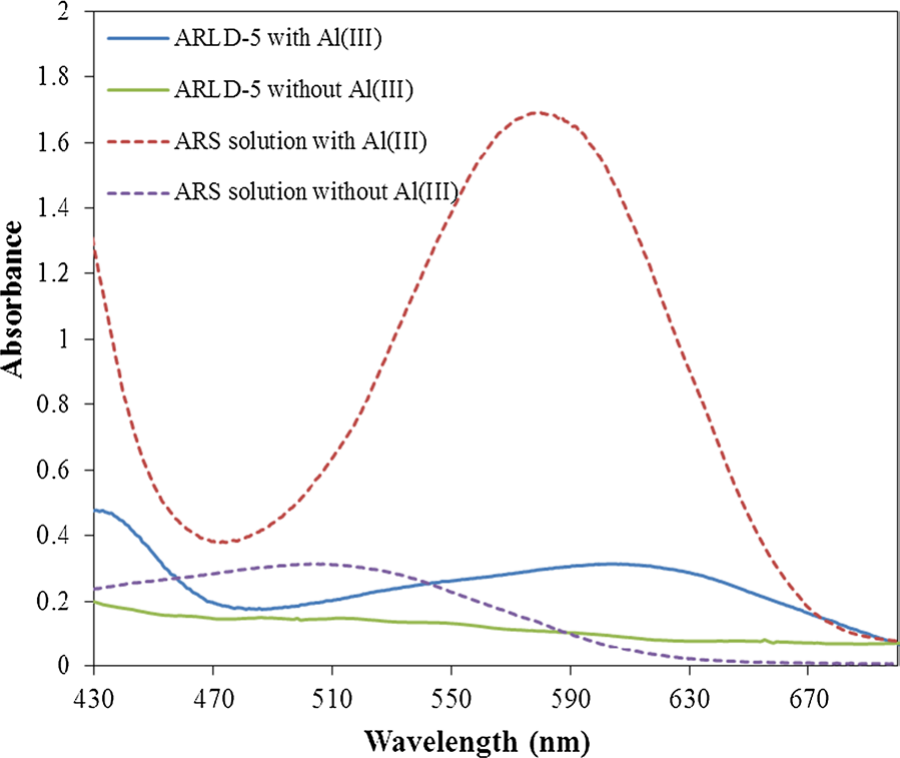
The UV–Vis spectrophotometry test of ARS solutions (10^–4^ M) in deionized water compared to modified solid electrodes before and after adding 120 μM Al^3+^

To better understand the electrochemical mechanism on the electrode surface, UV–Vis spectrophotometry investigation was performed on the electrode surface before and after adding 120 μM of Al^3+^ ion and performing a CV test. A.R.S. solution (10^–4^ M) in deionized water was also prepared. 120 μM of Al^3+^ ion was added to the solution and mixed well. The UV–Vis spectrophotometry test was then taken from both solutions. The obtained UV–Vis spectrophotometry results from the samples are shown in Fig. 12.

According to the results of Fig. 12, when the alumin**i**um ion was added to the ARS solution, we see a sharp peak at a maximum absorption wavelength of 577 nm, indicating an increase in the absorption wavelength (redshift) after the formation of the ARS-Al^3+^ complex in the ARS solution. When Al^3+^ ion reacted with the ARS molecule, the hydroxyl (–OH) functional group in the ARS molecule which acted as an auxochrome group has affected the shifting of carbonyl (C=O) chromophore peak to a longer wavelength at 577 nm as well as increasing the chromogen absorbance intensity [24]. The interesting point is that the absorption diagram of the ARLD-5 after adding the aluminium ion to the electrolyte solution and testing CV, a sharp peak at 590 nm absorption wavelength indicates that the formation of the ARS-Al^3+^ complex on the surface of the solid electrode is confirmed. In the study of Supian et al. [24], the wavelength of maximum absorption of the ARS-Al^3+^ complex was measured at 488 nm.

### Sensor Stability

Moreover, ten replicate measurements of 0.2 μM Al^3+^ on the modified electrode yielded a reproducible current and maximum fluorescence intensity with the relative standard deviation (R.S.D.) of 2.5% and 2.6%, respectively; also, six independent modified electrodes were prepared using the same procedure and used for determination of Al^3+^ (0.2 μM) with the (RSD) of 1.9%, demonstrating the excellent repeatability and reproduce ability of the electrochemical sensor. The long-term and too high flexibility were investigated by measuring the current response of 0.2 μM Al^3+^ after bending test (in a cylindrical manner to a 90° angle) of modified ITO/PET electrode for 90 times during 1 month and the per cent recovery was 96%, indicating the long lifetime stability of the modified flexible electrode.

**Fig. 13:**
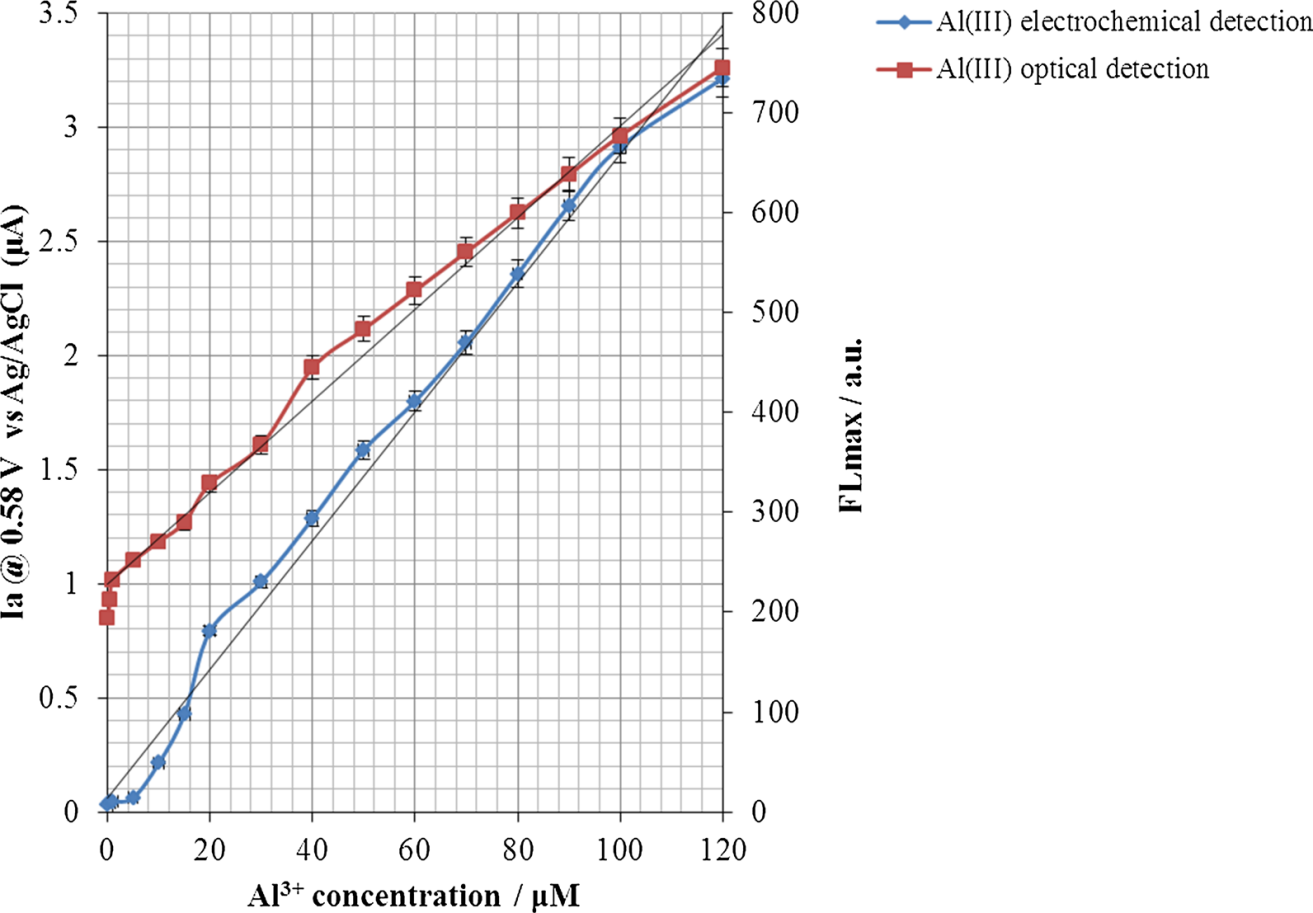
The electrochemical /optical response changes of the electrochemical sensor to the changes in Al^3+^ concentration

The results of Fig. 13 represent the electrochemical and optical response changes of the electrochemical sensor designed to the changes in the aluminium ion concentration, which shows a perfect and significant fit between the electrochemical and optical response of this type of sensor against the target molecule.

The detection limit in this work is compared to previous works so that the details are presented in Table 3.

**Table 3:** Comparison of the present sensor system and other electrochemical Al^3+^ detection methods

Chelators used	Chelator modification method	Method based on	Detection limit	References
Alizarin red S	LbL assembly of (A.R.S./LDHs)_n_ on ITO/PET	CV	10.1 nM	Present system
Alizarin red S	LbL assembly of (A.R.S./LDHs)_n_ on ITO/PET	Spectrofluorimetry	23 nM	Present system
Alizarin red	Drop coating	ASV	19.21 μM	[55]
Pyrocatechol violet	Adsorption	ASV	0.26 μM	[56]
8-Hydroxy-quinoline	Adsorption	ASV	4 ppm	[51]
Mercaptosuccinic acid	Self-assembly onto gold nanoparticles modified S.P.E	CV	37 nM	[38]
p-((8-hydrocycloquiboline)azo) benzenethiol	Self-assembly onto a gold electrode	Potentiometry	8.32 × 10^–3^ nM	[57]
Haematoxylin with graphite powder and paraffin wax	blending	Voltammetry and EIS	100 nM	[58]
Alizarin red S	Sol–gel on glassy carbon electrode	DPV	80 nM	[59]
Alizarin red	Polymerization with PVA-SbQ	Amperometry	25 μM	[60]

Other methods summarized in Table 3, based on the ASV technique, usually require longer detection times and repeatable electrode polishing step at each measurement. On the other hand, the electrochemical sensor designed in this study has a simple design system and, in addition to an electrochemical signal that accurately detects tiny amounts of target molecules quantitatively, also has an optical signal. As the optical signal changes in the spectrofluorimetry test, the application of the sensor is very convenient for biologists. This design structure is considered very suitable for future biotechnology applications.

## Conclusion

In this study, a novel flexible ITO/PET-based electrochemical sensor was designed and constructed so that by modifying its surface by (ARS/LDHs)_n_ matrix grid by LbL assembly technique, a sensitive and selective sensor was obtained to detect Al^3+^. The results of this study showed that the LbL assembly of LDH nanoplatelets along with the electroactive dye molecules (ARS), up to five cycles, formed a conductive matrix network with uniform morphology and topology. The minimum sheet resistance with the high electrocatalytic function was obtained in the fifth cycle of the LbL assembly. In this electrochemical sensor, both electrochemical and optical methods were detected with the high sensitivity of Al^3+^, so that in a CV-based electrochemical method, the lower detection limit of 10.1 nM with a broad linear range [0.2–120 μM] was obtained than fluorescence-based optical method. Although the electrochemical technique had a sensitivity of about 2.27 times more than an optical method in this sensor, due to the resolution of distinct optical response in different concentrations of Al^3+^, the optical applications will be an interest of biologists for biotechnological diagnostics.

## Data Availability

All data supporting the conclusions of this article are included within the article.

## Abbreviations

L.D.H.: Layered double hydroxides
LbL: Layer-by-layer
A.R.S: Alizarin red S
NCs: Nanoclusters
ITO/PET: ITO-coated polyethylene terephthalate
NALD: Naphthalimide dye/LDH nanoplatelets matrix
ICP-AES: Inductively coupled plasma atomic emission spectroscopy
ICP-MS: Inductively coupled plasma mass spectrometry
GF-AAS: Graphite furnace atomic absorption spectroscopy
HR-CS: High-resolution continuum source flame atomic absorption spectrometer
FAAS: Flame atomic absorption spectrometry
ASV: Adsorptive stripping voltammetry
DPV: Differential pulse voltammetry
EIS: Electrical impedance spectroscopy
CV: Cyclic voltammetry
PZC: Point of zero charges
SEM: Scanning electron microscope
AFM: Atomic force microscopy
SPE: Solid phase extraction

## References

[1] Nagendra B, Mohan K, Gowd EB (2015). Polypropylene/layered double hydroxide (L.D.H.) nanocomposites: influence of L.D.H. particle size on the crystallization behaviour of polypropylene. ACS Appl Mater Interfaces.

[2] Weidan N, Zhengyi Q, Xueqian C, Xingguang S (2018). A turn-on fluorescent probe for sensitive detection of sulfide anions and ascorbic acid by using sulfanilic acid and glutathione functionalized graphene quantum dots. Sens Actuators B Chem.

[3] Alibakhshi E, Ghasemi E, Mahdavian M, Ramezanzadeh B (2016). Corrosion inhibitor release from Zn-Al-[PO43-]-[CO32-] layered double hydroxide nanoparticles. Prog Color Color Coat.

[4] Jayakumar K, María Belén C, Dharuman V, Huangxian J, Ramendra SD, Yangping W (2018). One-step electrodeposition-assisted layer-by-layer assembly of gold nanoparticles and reduced graphene oxide and its self-healing three-dimensional nanohybrid for an ultrasensitive D.N.A. sensor. Nanoscale.

[5] Guangwei G, Jie L, Zaochuan G, Shaojun C, Shiguo C, Haipeng Y (2017). Self-Assembling of electrochemical glucose biosensor with bacteriostatic materials via layer-by-layer method. J Electrochem Soc.

[6] Run-Min S, Zhan-Hong L, Peng-Ju W, Xue-Ling Z, Cheng C, Zhi-Gang Z (2018). Flexible hydrogen peroxide sensors based on platinum modified free-standing reduced graphene oxide paper. Appl Sci.

[7] Yuan T, Pu L, Huang Q, Zhang H, Li X, Yang H (2014). An effective methanol-blocking membrane modified with graphene oxide nanosheets for passive direct methanol fuel cells. Electrochim Acta.

[8] Kong XG, Rao XY, Han JB, Wei M, Duan X (2010). Layer-by-layer assembly of bi-protein/layered double hydroxide ultrathin film and its electrocatalytic behaviour for catechol. Biosens Bioelectron.

[9] Chen X, Fu CL, Wang Y, Yang WS, Evans DG (2008). Direct electrochemistry and electrocatalysis based on a film of horseradish peroxidase intercalated into Ni–Al layered double hydroxide nanosheets. Biosens Bioelectron.

[10] Taviot-Gueho C, Prevot V, Forano C, Renaudin G, Mousty C, Leroux F (2017). Tailoring Hybrid layered double hydroxides for the development of innovative applications. Adv Funct Mater.

[11] Chen H, Hu L, Chen M, Yan Y, Wu L (2014). Nickel-Cobalt layered double hydroxide nanosheets for high-performance supercapacitor electrode materials. Adv Funct Mater.

[12] Li S, Lu J, Wei M, Evans DG, Duan X (2010). Tris(8-hydroxyquinoline-5-sulfonate) aluminium intercalated Mg–Al layered double hydroxide with blue luminescence by hydrothermal synthesis. Adv Funct Mater.

[13] Tian R, Zhang S, Li M, Zhou Y, Lu B, Yan D, Wei M, Evans DG, Duan X (2015). Localization of Au nanoclusters on layered double hydroxides nanosheets: confinement-induced emission enhancement and temperature-responsive luminescence. Adv Funct Mater.

[14] Marrani AG, Coico AC, Giacco D, Zanoni R, Motta A, Schrebler R, Dini Girolamo DD, Dalchiele EA (2019). Flexible interfaces between reduced graphene oxide and indium tin oxide/polyethylene terephthalate for advanced optoelectronic devices. ACS Appl Nano Mater.

[15] Dong H, Wu Z, Jiang Y, Liu W, Li X, Jiao B, Abbas W, Hou X (2016). A flexible and thin graphene/silver nanowires/polymer hybrid transparent electrode for optoelectronic devices. ACS Appl Mater Interfaces.

[16] Chen Y, Zhang Y, Liang Z, Cao Y, Han Z, Feng X (2020). Flexible inorganic bioelectronics. npj Flex Electron.

[17] Li R, Qi H, Ma Y, Deng Y, Liu S, Jie Y, Jing J, He J, Zhang X, Wheatley L, Huang C, Sheng X, Zhang M, Yin L (2020). A flexible and physically transient electrochemical sensor for real-time wireless nitric oxide monitoring. Nat Commun.

[18] Chang WY, Fang TH, Yeh SH, Lin YC (2009). Flexible electronics sensors for tactile multi-touching. Sensors (Basel).

[19] Lajevardi Esfahani S, Rouhani S, Ranjbar Z (2019). Electrochemical solid-state nanosensor based on a dual amplification strategy for sensitive detection of (FeIII-dopamine). Electrochim Acta.

[20] Ding F, Diao JX (2011). Characterization of alizarin red S binding sites and structural changes on human serum albumin: a biophysical study. J Hazard Mater.

[21] Wang Z, Liu X, Baeyens WRG (2008). Copper(II)−alizarin red S complex as an efficient chemiluminescent probe for the detection of human serum proteins after polyacrylamide gel electrophoresis. J Proteome Res.

[22] Wang LL, Qiao J, Liu HH (2014). Ratiometric fluorescent probe based on gold nanoclusters and alizarin red-boronic acid for monitoring glucose in brain microdialysate. Anal Chem.

[23] Halls JE, Ahn SD, Jiang D (2013). Proton uptake vs. redox driven release from metal–organic-frameworks: alizarin red S reactivity in UMCM-1. J Electroanal Chem.

[24] Supian SM, Ling TL, Heng LY, Chong KF (2013). Quantitative determination of Al(III) ion by using alizarin red S, including its microspheres optical sensing material. Anal Methods.

[25] Liang P, Yang L, Hu B, Jiang Z (2003). ICP-AES detection of ultratrace aluminium (III) and chromium(III) ions with a microcolumn preconcentration system using dynamically immobilized 8-hydroxyquinoline on TiO_2_ nanoparticles. Anal Sci.

[26] Zheng H, Gao X, Song L (2011). Preconcentration of trace aluminium (III) ion using a nanometer-sized TiO_2_-silica composite modified with 4-aminophenylarsonic acid, and its determination by ICP-OES. Microchim Acta.

[27] Djerahov L, Vasileva P, Karadjova I, Kurakalva RM, Aradhi KK (2016). Chitosan film loaded with silver nanoparticles—sorbent for solid phase extraction of Al(III), Cd(II), Cu(II), Co(II), Fe(III), Ni(II), Pb(II) and Zn(II). Carbohydr Polym.

[28] Mashhadizadeh MH, Amoli-Diva M (2013). Atomic absorption spectrometric determination of Al^3+^ and Cr^3+^ after preconcentration and separation on 3-mercaptopropionic acid modified silica coated-Fe_3_O_4_ nanoparticles. J Anal At Spectrom.

[29] Ciftci H, Er C, Ozkan M (2016). Determination of aluminium from water samples and dialysis fluids after separation/preconcentration on Duolite XAD-761 polymeric resin. Desalin Water Treat.

[30] Yalçınkaya O, Erdoğan H, Çiftçi H, Türker AR (2012). Preconcentration of aluminum on nano ZrO_2_/B_2_O_3_ and its determination by flame atomic absorption spectrometry. Spectrosc Lett.

[31] Suah FBM, Ahmad M, Heng LY (2014). Highly sensitive fluorescence optode for aluminium(III) based on non-plasticized polymer inclusion membrane. Sens Actuators B Chem.

[32] Muk NS, Narayanaswamy R (2006). Fluorescence sensor using a molecularly imprinted recognition receptor for the detection of aluminium ions in aqueous media. Anal Bioanal Chem.

[33] Supian S, Ling T, Heng lY, Chong KF (2013). Quantitative determination of Al(III) ion by using Alizarin Red S, including its microspheres optical sensing material. Anal Methods.

[34] Mendecki L, Granados-Focil S, Jendrlin M, Mold M, Radu A (2020). Self-plasticized, lumogallion-based fluorescent optical sensor for the determination of aluminium (III) with ultra-low detection limits. Anal Chim Acta.

[35] Ahmad M, Narayanaswamy R (1995). A flow-cell optosensor for monitoring aluminium(III) based on immobilised eriochrome cyanine R (ECR) and reflectance spectrophotometry. Sci Total Environ.

[36] Castañeda-Loaiza V, Díaz-de-Alba MI, Granado-Castro MD, Galindo-Riaño MD, Casanueva-Marenco MJ (2019). Disposable optical sensor for Al(III) ions determination by coupled colorimetric solid-phase extraction-reflectance spectroscopy in leachates from cookware, antacids and hygienic care products. Talanta.

[37] İşlek Y, Henden E (2020). Selective and sensitive fluorimetric determination of aluminium in water samples and dialysis solutions after preconcentration using a novel Ni/NixB nano adsorbent. J Anal Chem.

[38] See WP, Heng LY, Nathan S (2015). Highly Sensitive Aluminium(III) ion sensor based on a self-assembled monolayer on a gold nanoparticles modified screen-printed carbon electrode. Anal Sci.

[39] Wu J, Chen X, Wang Q, Bian Y, Zhang K, Sheng Z, Jin J, Yang M, Dai P, Fu X, Chang W, Xie C (2018). Organic–inorganic‐hybrid‐enhancement electrochemical sensor for determination of Cu (II) in river water. Electroanalysis.

[40] Alibakhshi E, Ghasemi E, Mahdavian M, Ramezanzadeh B, Farashi S (2017). Active corrosion protection of Mg–Al–PO_4_^3−^ L.D.H. nanoparticle in silane primer coated with epoxy on mild steel. J Taiwan Inst Chem Eng.

[41] Liu M, Lv G, Mei L, Wei Y, Liu J, Li Z, Liao L (2017). Fabrication of AO/LDH fluorescence composite and its detection of Hg^2+^ in water. Sci Rep.

[42] Xu ZP, Zeng QH, Lu GQ, Yu AB (2006). Inorganic nanoparticles as carriers for efficient cellular delivery. Chem Eng Sci.

[43] Li SP, Hou WG, Han SH, Li LF, Zhao WA (2003). Studies on intrinsic ionization constants of Fe–Al–Mg hydrotalcite-like compounds. J Colloid Interface Sci.

[44] Hu WG, Sun DJ, Zhang CG, Su YL (2001). Studies on zero point of charge and permanent charge density of Mg–Fe hydrotalcite-like compounds. Langmuir.

[45] Sathish RS, Kumar MR, Rao GN, Kumar KA, Janardhana C (2007). A water-soluble fluorescent fluoride ion probe based on alizarin red S-Al(III) complex. Spectrochim Acta Part A.

[46] Rouhani S, Nahavandifard F (2014). Molecular imprinting-based fluorescent optosensor using a polymerizable 1,8-naphthalimide dye as a fluorescence functional monomer. Sens Actuators B Chem.

[47] Srujana P, Sudhakar P, Radhakrishnan TP (2018). Enhancement of fluorescence efficiency from molecules to materials and the critical role of molecular assembly. J Mater Chem C.

[48] Turcanu A, Bechtold T (2011). pH Dependent redox behaviour of Alizarin Red S (1,2-dihydroxy-9,10- anthraquinone-3-sulfonate)—cyclic voltammetry in presence of dispersed vat dye. Dyes Pigments.

[49] Fujikawa H, Yamaguchi S, Matsui K (2018). Spectroscopic study of alizarin and alizarin red adsorbed on anodic aluminum oxide films. Trans Mater Res Soc Jpn.

[50] Fain VY, Zaitsev BE, Ryabov MA (2004). Metal complexes with alizarin and alizarin red S: electronic absorption spectra and structure of ligands. Russ J Coord Chem.

[51] Stryjewska E, Rubel S (1991). Adsorptive stripping voltammetry for determination of trace amounts of aluminium with calmagite. Electroanalysis.

[52] O'sullivan B (1997) PhD thesis on electroanalysis of aluminum. University of Canterbury, pp 130–160

[53] Dos Santos T, Aucélio R, Campos R (2003). Spectrofluorimetric method for the determination of aluminum with alizarin red PS. Microchim Acta.

[54] Moore SA, Glenn KM, Palepu RM (2007). Spectroscopic investigations on the interaction of Crystal Violet with nonionic micelles of Brij and Igepal surfactants in aqueous media. J Solut Chem.

[55] Downard AJ, Kipton H, Powell J, Xu S (1991). Voltammetric determination of aluminium(III) using a chemically modified electrode. Anal Chim Acta.

[56] Vukomanovic DV, Page JA, Vanloon GW (1991). Voltammetric determination of Al(III) with adsorptive preconcentration of the pyrocatechol violet complex. Can J Chem.

[57] Mashhadizadeh MH, Talemi RP (2011). Used gold nanoparticles as an on/off switch for response of a potentiometric sensor to Al(III) or Cu(II) metal ions. Anal Chim Acta.

[58] Shervedani RK, Rezvaninia Z, Sabzyan H, Boeini HZ (2014). Characterization of gold-thiol-8 hydroxyquinoline self-assembled monolayers for selective recognition of aluminium ion using voltammetry and electrochemical impedance spectroscopy. Anal Chim Acta.

[59] Di J, Bi S, Yang T, Zhang M (2004). Voltammetric determination of aluminium (III) using a reagent less sensor fabricated by sol-gel process. Sens Actuators B Chem.

[60] Chang SC (2010). Alizarin red S modified electrochemical sensors for the detection of aluminium ion. J Korean Sens Soc.

